# Clumps of Mesenchymal Stem Cell/Extracellular Matrix Complexes Generated with Xeno-Free Conditions Facilitate Bone Regeneration via Direct and Indirect Osteogenesis

**DOI:** 10.3390/ijms20163970

**Published:** 2019-08-15

**Authors:** Souta Motoike, Mikihito Kajiya, Nao Komatsu, Susumu Horikoshi, Tomoya Ogawa, Hisakatsu Sone, Shinji Matsuda, Kazuhisa Ouhara, Tomoyuki Iwata, Noriyoshi Mizuno, Tsuyoshi Fujita, Makoto Ikeya, Hidemi Kurihara

**Affiliations:** 1 Department of Periodontal Medicine, Graduate School of Biomedical and Health Sciences, Hiroshima University, Hiroshima 734-8553, Japan; 2 Department of Clinical Application, Center for iPS Cell Research and Application, Kyoto University, Kyoto 606-8507, Japan

**Keywords:** xeno-free, scaffold-free, bone regeneration, MSCs, 3D culture

## Abstract

Three-dimensional clumps of mesenchymal stem cell (MSC)/extracellular matrix (ECM) complexes (C-MSCs) consist of cells and self-produced ECM. We demonstrated previously that C-MSCs can be transplanted into bone defect regions with no artificial scaffold to induce bone regeneration. To apply C-MSCs in a clinical setting as a reliable bone regenerative therapy, the present study aimed to generate C-MSCs in xeno-free/serum-free conditions that can exert successful bone regenerative properties and to monitor interactions between grafted cells and host cells during bone healing processes. Human bone marrow-derived MSCs were cultured in xeno-free/serum-free medium. To obtain C-MSCs, confluent cells that had formed on the cellular sheet were scratched using a micropipette tip and then torn off. The sheet was rolled to make a round clump of cells. Then, C-MSCs were transplanted into an immunodeficient mouse calvarial defect model. Transplantation of C-MSCs induced bone regeneration in a time-dependent manner. Immunofluorescence staining showed that both donor human cells and host mice cells contributed to bone reconstruction. Decellularized C-MSCs implantation failed to induce bone regeneration, even though the host mice cells can infiltrate into the defect area. These findings suggested that C-MSCs generated in xeno-free/serum-free conditions can induce bone regeneration via direct and indirect osteogenesis.

## 1. Introduction

Mesenchymal stem cells (MSCs), a class of adult stem cells, are attractive candidates for tissue regenerative therapy because of their relative ease of isolation, self-renewing properties, and multipotency [1,2]. In addition, their trophic factor production and immunomodulatory capacity make MSCs a promising treatment option for various types of disease [3,4]. In particular, bone marrow-derived MSCs are well-utilized stem cells for bone regeneration, both in basic research and in clinical practice [5,6]. Compared with the injection of isolated cell suspensions, combining MSCs with biomaterial scaffolds is a routinely employed strategy for bone regenerative therapy. Indeed, such tissue engineering approaches by seeding cells into an artificial scaffold have shown promising clinical results [7]. Nevertheless, the usage of artificial scaffolds may present some complications such as biodegradability and host unfavorable inflammatory reactions. Moreover, the process of combining MSCs and biomaterials requires enzymatic digestion of the cells, which degrades extracellular matrix (ECM) proteins and reduces cellular function.

To avoid these problems, we recently developed clumps of MSCs/ECM complexes (C-MSCs), which consisted of cells and self-produced ECM [8]. Three-dimensional-cultured C-MSCs can be transplanted into bone defects with no artificial scaffold to induce bone regeneration. In addition, C-MSCs cultured with osteoinductive medium induced more-effective bone regeneration in rat calvaria [8] and in a beagle dog periodontal tissue defect model [9]. We have also reported that interferon (IFN)-γ treatment of human C-MSCs elevated immunomodulatory enzyme indoleamine 2,3-dioxygenease (IDO) expression in vitro, and its xenotransplantation into a mouse calvarial defect model suppressed immune rejection by T cells and induced successful bone regeneration [10]. Briefly, C-MSCs, which can be regulated their cellular function before implantation, could be promising cell transplantation therapy avoiding the limitation of artificial scaffolds. However, to apply C-MSCs in a clinical setting as a reliable bone regenerative therapy, some obstacles have yet to be overcome.

One of the drawbacks of C-MSCs is the usage of fetal bovine serum (FBS). Although FBS contains a cocktail of growth factors, cell attachment proteins, and other important biomolecules, its clinical application raises serious concerns related to possible microbiological contamination and the potential transmission of animal disease to humans [11,12]. Moreover, the precise composition in FBS is still unclear and there is high variability between batches [13,14]. In the context of establishing standardized culture conditions for safe and reliable clinical applications of C-MSCs, xeno-free/serum-free medium that can guarantee a well-defined composition, high stability, and reduced risk of contamination should be utilized.

To develop transplantation therapy with C-MSCs based on scientific evidence, another issue to be aware of is the molecular mechanism involved in the bone reconstructive process induced by C-MSCs. Of note, it was reported that MSC transplantation induces bone repair based on direct donor cells osteogenic differentiation effects [15] or based on its indirect paracrine effects that stimulate host cells tissue reconstruct capacity [16,17]. Even though we reported that transplantation of C-MSCs successfully induced bone regeneration, it remains unclear whether such bone reconstruction is due to donor direct or host indirect osteogenesis. Moreover, C-MSC implantation can provide not only multipotent cells but also intact ECM proteins that can have a role in tissue reconstruction [18]. Briefly, it is of great interest to understand whether ECM proteins produced by cells in C-MSCs contribute to the bone healing process.

Accordingly, to facilitate the future clinical application of C-MSCs, this study aimed to generate C-MSCs in xeno-free/serum-free conditions that can exert successful bone regenerative properties and investigate how donor MSCs, grafted ECM proteins, and host cells are involved in the bone reconstruction process induced by C-MSCs in a SCID mouse calvarial defect model. 

## 2. Results

### 2.1. Generation of 3D-Culture C-MSCs in Xeno-Free Conditions

**Figure 1:**
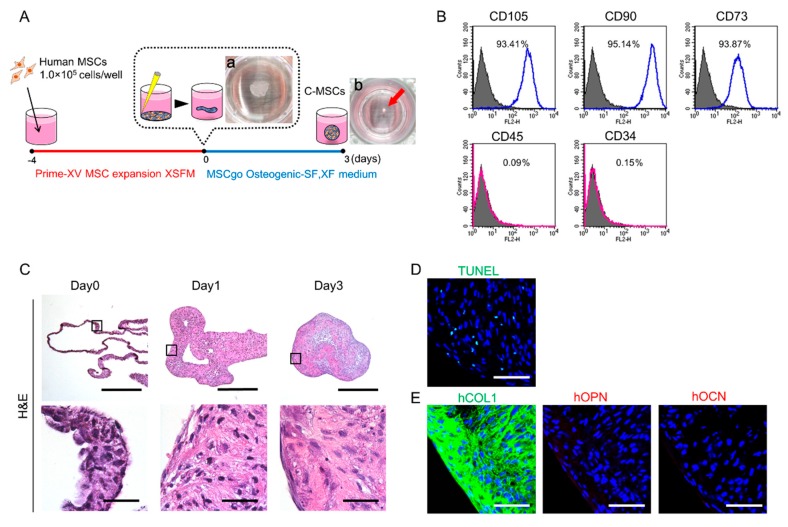
Generation of mesenchymal stem cell (MSC)/extracellular matrix (ECM) complexes (C-MSCs) in xeno-free conditions. (**A**) Schematic image of C-MSCs generation in xeno-free culture conditions. a: Macroscopic image of cellular sheet on day 0. b: Macroscopic image of cell clumps on day 3. (**B**) Cells cultured with Prime-XV MSC expansion XSFM for 4 days were isolated and their surface marker expression levels were monitored by flow cytometry. Open histograms with blue or red lines indicate CD105-, CD90-, CD73-, CD45-, or CD34-positive cells. The isotype-matched control IgG is shown as a solid histogram. (**C**) C-MSCs were cultured in floating conditions until the end of the culture period and were stained with H&E. The upper panels show lower magnification (Bar = 500 µm), and magnified images in the boxed regions are shown in the lower panels (Bar = 50 µm). (**D** and **E**) C-MSCs were cultured for 3 days in xeno-free conditions. Serial sections were stained with TUNEL (green) (**D**) or bone matrix-related proteins, including COL1 (green), OPN (red), and OCN (red) (**E**), respectively. Nuclei (blue) were counter-stained with DAPI. Bar = 50 µm. All graphs and images are representative of three independent experiments.

Three-dimensional C-MSCs were generated in xeno-free conditions as described in the Materials and Methods section. Briefly, to obtain MSCs/ECM complexes, confluent MSCs treated with Prime-XV MSC expansion XSFM for 4 days were scratched using a micropipette tip (Figure 1A-a). The sheets of MSCs/ECM complexes were detached from the culture plate and maintained in MSCgo Osteogenic differentiation medium for 3 days. Then, we obtained C-MSCs as round cell clumps that consisted of cells and self-produced ECM (Figure 1A-b). To investigate whether 3D culture with xeno-free condition affect MSC markers expression in the cells, FACS analysis was conducted. At the beginning of C-MSC culture with xeno-free condition, we confirmed that more than 93% of the cells expressed MSC-positive markers, such as cluster of differentiation (CD)73, CD90, and CD105, although the number of cells expressing negative markers including CD34 and CD45 were very few in the sheet shape of MSCs/ECM complexes (Figure 1B), suggesting that 3D floating culture C-MSCs with xeno-free condition maintain the MSCs property. HE staining demonstrated that the cells and ECM complexes rolled up to make round cell clumps in a time-dependent manner (Figure 1C). Critical cell apoptosis was not observed in the generated C-MSCs (Figure 1D). Immunofluorescence analysis showed that among the major extracellular bone matrix proteins, collagen (COL)1 was mainly produced in C-MSCs but not osteopontin (OPN) or osteocalcin (OCN) (Figure 1D).

### 2.2. Implantation of C-MSCs Generated in Xeno-Free Conditions with No Artificial Scaffold Can Induce Mouse Calvarial Bone Regeneration

**Figure 2:**
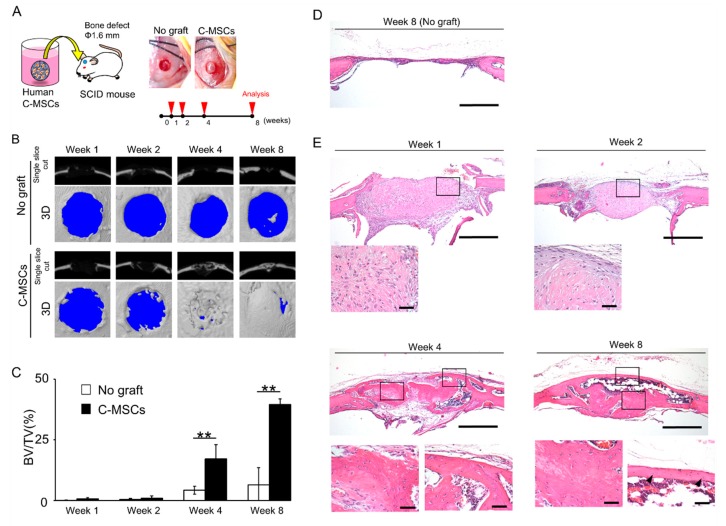
Implantation of C-MSCs generated in xeno-free conditions with no artificial scaffold can induce mouse calvarial bone regeneration. (**A**) Study design for the in vivo experiment. C-MSCs were transplanted into a SCID mouse calvarial defect 1.6 mm in diameter with no artificial scaffold. No graft group was set as a control. (**B**) Representative µCT images at 1, 2, 4, and 8 weeks after surgery. (**C**) Ratio of the segmented bone volume (BV) to the total volume (TV) of the defect region at 1, 2, 4, and 8 weeks following surgery. Data are mean ± SD of four mice per group. **p < 0.01 for each respective time point. (**D**–**H**) Animals were sacrificed at 1, 2, 4, and 8 weeks after surgery and the calvarial bones were fixed. Coronal sections were obtained and stained with H&E. (**D**) No graft group at 8 weeks after surgery. Bar = 500 µm. (**E**) C-MSCs grafted group at 1, 2, 4, and 8 weeks following transplantation. Bar = 500 µm. Bottom panels are magnifications of the boxed regions. Bar = 50 µm. The photographs are representative of four independent experiments.

To test whether C-MSCs generated in xeno-free conditions possess bone regenerative capacity, C-MSCs were directly transplanted into a SCID mouse calvarial defects model without any artificial scaffold (Figure 2A). Micro-CT single cut and 3D reconstructed images showed unsuccessful bone regeneration in the no graft groups during the observation period (Figure 2B). Transplantation of C-MSCs induced successful bone regeneration in a time-dependent manner (Figure 2B,C). Consistent with this micro-CT data, new bone formation was not observed, and only thin soft fibrous tissue connected the edges of the defect area in the no graft group (Figure 2D). On the other hand, at 1 week after transplantation of C-MSCs, abundant connective tissue including a number of cells, which could be presumed to be the grafted C-MSCs, were found in the defect area and it was condensed after 2 weeks (Figure 2E). In addition, new bone formation occurred slightly from the edge of the defect, and the lesion area was covered with periosteum (Figure 2E). Notably, at 4 weeks after transplantation, immature bone-like tissue was formed at the center of the defect, which was surrounded with thin new bone extending from the peripheries of the defect (Figure 2E). Finally, a new mass of bone including bone marrow-like structure bridged the defect gap after 8 weeks (Figure 2E). These findings clearly suggested that C-MSCs generated in xeno-free conditions have bone regenerative capacity.

### 2.3. Transplanted Human Donor and Mouse Host Cells Contribute to Bone Reconstruction Induced by Transplantation of C-MSCs

**Figure 3:**
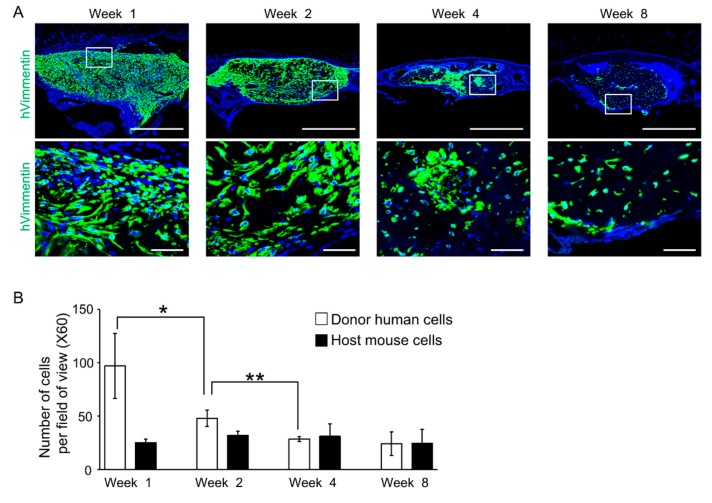
Transplanted human donor and mouse host cells contribute to the bone reconstruction induced by transplantation of C-MSCs. (**A**) Animals were sacrificed at 1, 2, 4, and 8 weeks after surgery and the calvarial bones were fixed. Coronal sections were obtained and immunostained with anti-human Vimentin antibody (green). Nuclei (blue) were counter-stained with DAPI. Upper panels show lower magnification. Bar = 500 µm. Bottom panels are magnifications of the boxed regions. Bar = 50 µm. (**B**) The periphery (left and right), mid (left and right), and center in the bone defect region from each group were used for counting of human vimentin-positive and -negative cells. Results are expressed as means ± SD of the five views tested for each group. * *p* < 0.05, ** *p* < 0.01: Values differ significantly. All graphs and images are representative of four independent experiments.

To investigate donor and host cells’ behavior in the process of bone regeneration induced by C-MSCs, immunohistochemistry using anti-human vimentin antibodies was conducted. At 1 and 2 weeks after transplantation, the defects were filled with human vimentin-positive cells, suggesting grafted human C-MSCs. Then, the number of human cells decreased in a time-dependent manner (Figure 3A,B), although the newly formed bone filling the defects expressed human vimentin, confirming that progeny of the transplanted C-MSCs survived and differentiated into osteogenic cells for at least 8 weeks (Figure 3A,B). On the other hand, human vimentin-negative cells, suggesting host mouse cells, were observed in the new bone covering the defect at 4 and 8 weeks after implantation (Figure 3A). These findings suggested that donor human cells and host mouse cells contributed to bone reconstruction induced by transplantation of C-MSCs.

### 2.4. Transplantation of C-MSCs Induces Bone Regeneration via Direct and Indirect Osteogenesis in a SCID Mouse Calvarial Defect Model

**Figure 4:**
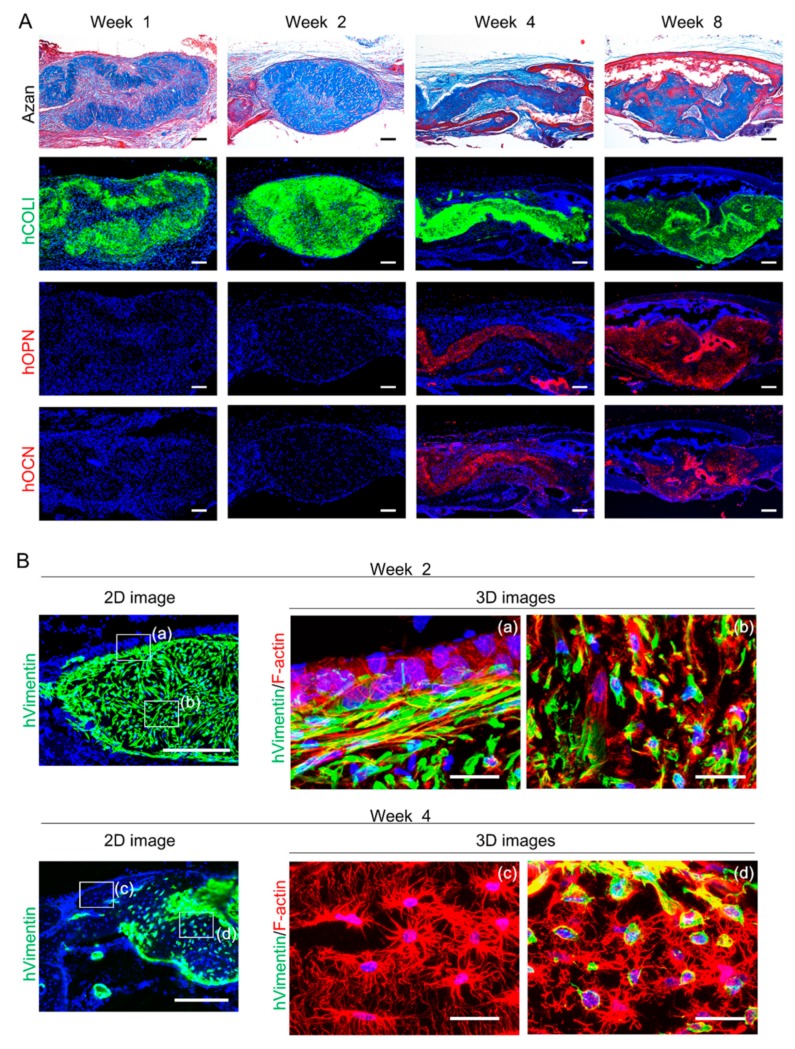
Transplantation of C-MSCs induces bone regeneration via direct and indirect osteogenesis in a SCID mouse calvarial defect model. (**A** and **B**) Animals were sacrificed at 1, 2, 4, and 8 weeks after surgery and the calvarial bones were fixed. Semi-serial sections were obtained and stained with AZAN and immunostained with anti-human COLI, anti-human OPN, and anti-human OCN antibodies, as indicated. Nuclei were counterstained with DAPI for immunostaining. Upper panels show lower magnifications. Bar = 200 µm. Bottom panels are magnifications of the boxed regions. Bar = 50 µm. (**B**) Animals were sacrificed at 2 and 4 weeks after surgery and the calvarial bones were fixed. Sections obtained using a cryostat were stained with anti-human vimentin antibody and phalloidin, as indicated. Nuclei were counterstained with DAPI. Left panels are lower magnification images showing vimentin and nuclei. Bar = 250 µm. Right panels are 3D immunofluorescence images of the boxed region of lower magnification images labeled with (a), (b), (c), and (d) showing human vimentin (green), F-actin (red), and nuclei (blue). Bar = 25 µm. The photographs are representative of four independent experiments.

Based on findings that both donor and host cells contribute to bone regeneration, the quality of newly formed bone was next assessed using AZAN and immunofluorescence staining for human bone matrix proteins, including COL1, OPN, and OCN. At 1 and 2 weeks after transplantation of C-MSCs, connective fibrous tissues were visualized using AZAN staining (Figure 4A). This connective tissue displayed positivity for human COL1, confirming the progeny of grafted C-MSCs, whereas human non-collagenous bone matrix proteins, OPN and OCN were not detectable in the extracellular environment (Figure 4A). Of note, at 4 and 8 weeks after implantation, Azan staining showed that immature bone had started to form at the center of the defect and it matured in a time-dependent manner, indicated by an increasing the red-colored zone (Figure 4A). Moreover, the new bone at the central area was covered with more mature bone extending from the edge of the defect, which clearly showed a red color using Azan staining (Figure 4A). Then, human COL1, OPN, and OCN were expressed in the newly formed bone at the center of the site at 4 and 8 weeks after transplantation, confirming the contribution of bone matrix proteins secreted from donor human cells for bone regeneration. These findings suggested the possibility that during the 2-week window from 2–4 weeks after the transplantation, donor direct and host indirect osteogenesis occurred, which resulted in the induction of successful bone regeneration. Accordingly, because the appearance of osteocytes is the hallmark of the genuine osteogenesis, we observed cellular morphology at 2 and 4 weeks after implantation using immunofluorescence 3D images for actin filaments. At 2 weeks after transplantation, human vimentin-positive donor cells showed a spindle fibroblastic shape inside the grafted C-MSCs, where host mouse cells were lacking (Figure 4B-(b)). At the interface between grafted C-MSCs and host tissue, human vimentin-positive cells expressed stress fiber formation implying matrix stiffness. In addition, on the layer of the human elongated cells, human vimentin-negative cells that formed cuboidal shapes, suggesting that host osteoblasts were observed (Figure 4B-(a)). At 4 weeks after transplantation of C-MSCs, many cells had formed dendrite processes, suggestive of osteocytes, were observed in the newly reconstructed bone. Notably, in new bone extending from the host tissue, human vimentin-negative osteocytes were observed (Figure 4B-(c)), whereas human vimentin-expressing osteocytes were connected to each other in the center of new bone that could have descended from grafted C-MSCs (Figure 4B-(d)). These findings clearly demonstrated that transplantation of C-MSCs induced bone regeneration via donor direct and host indirect successful osteogenesis.

### 2.5. Transplantation of Decell-C-MSCs Fails to Induce Bone Regeneration

**Figure 5:**
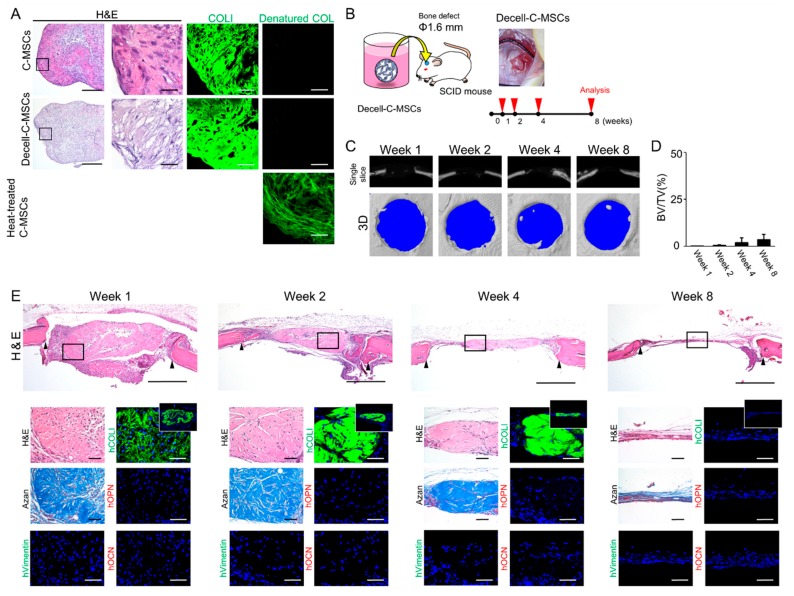
Transplantation of Decell-C-MSCs failed to induce bone regeneration. (**A**) C-MSCs, Decell-C-MSCs, and heat-treated C-MSCs were prepared as described in the Materials and Methods section. Semi-serial sections were stained with H&E and immunostained with anti-COLI antibody and 5-FAM conjugated-collagen hybridizing peptide, as indicated. Left panels show lower magnification. Bar = 200 µm. The second to fourth panels are magnifications of the boxed regions. Bar = 50 µm. (**B**) Study design for the in vivo experiment. Decell-C-MSCs were transplanted into a SCID mouse calvarial defect 1.6 mm in diameter with no artificial scaffold. (**C**) Representative µCT images at 1, 2, 4, and 8 weeks after surgery. (**D**) Ratio of the segmented bone volume (BV) to the total volume (TV) of the defect region at 1, 2, 4, and 8 weeks following surgery. Data are mean ± SD of four mice per group. (**E**) Animals were sacrificed at 1, 2, 4, and 8 weeks after surgery and the calvarial bones were fixed. Semi-serial sections were obtained and stained with H&E and AZAN and immunostained with anti-human vimentin, anti-human COLI, anti-human OPN, and anti-human OCN antibodies, as indicated. Nuclei were counterstained with DAPI for immunostaining. Upper panels show lower magnification. Bar = 200 µm. Bottom panels are magnifications of the boxed region. Bar = 50 µm. White boxes indicate human COL1 expression in the whole defect area. The photographs are representative of four independent experiments.

C-MSCs consist of the cells and self-produced ECM, such as COL1. Accordingly, there is the possibility that the ECM but not the grafted cells play a role in bone regeneration induced by transplantation of C-MSCs. To test this possibility, we generated decellularized ECM derived from C-MSCs (Decell-C-MSCs), as described in the Materials and Methods section. HE staining clearly demonstrated that neither nuclei stained with hematoxylin nor cytoplasmic components stained with eosin were observed in Decell-C-MSCs (Figure 5A). However, immunofluorescence analysis showed similar expression levels of human COL1 between Decell-C-MSCs and C-MSCs (Figure 5A). In addition, to confirm the quality of this COL1 in Decell-C-MSCs, 5-FAM conjugated collagen hybridizing peptide (CHP), which can detect denatured collagen fibers, was employed. CHP apparently hybridized with heat-treated C-MSCs, which were used as a positive control, although no signals were detected in C-MSCs or Decell-C-MSCs (Figure 5A). These findings suggested that Decell-C-MSCs retained intact COL1 but not cellular components. Then, Decell-C-MSCs were transplanted directly into a SCID mouse calvarial defect, as shown in Figure 5B. Micro-CT analysis demonstrated that implantation of Decell-C-MSCs completely failed to induce bone regeneration (Figure 5C,D). Histological analysis demonstrated that the defect was filled with fibrous connective tissues, which was composed of human COL1 but not OPN/OCN at 1 week after transplantation. However, the fibrous tissue could not maintain its shape and degraded in a time-dependent manner (Figure 5E). Of note, although human vimentin-negative host cells penetrated into the grafted Decell-C-MSCs, they had not started to produce any bone matrix and only formed thin soft tissue connecting the defect edges (Figure 5E). These findings clearly demonstrated that donor human cells were responsible for the bone regeneration induced by the transplantation of C-MSCs.

## 3. Discussion

This study demonstrated that transplantation of human C-MSCs generated in xeno-free conditions induced successful bone regeneration in a SCID mouse calvarial defect model. Part of the newly formed bone expressed bone matrix proteins provided from donor human cells. Otherwise, periphery of the new reconstructed bone that was mature and covered the grafted area consisted of only mice cells. In addition, donor human- and host mouse-derived osteocytes were also observed. These findings suggested that transplantation of C-MSCs induced donor direct and host indirect osteogenesis that cooperatively contributed to new bone reconstruction. Nowadays, the precise mechanism of bone regeneration by implanted MSCs is still controversial and not completely defined [19]. For example, Linero et al. reported that both adipose-derived MSCs and their conditioned medium transplantation with hydrogel into a rabbit jaw bone defect induced bone regeneration, although the donor cells completely disappeared in the healing process [20]. These findings clearly indicated the paracrine effect of MSCs. On the other hand, Li et al. showed that bone marrow-derived MSCs infused into femurs of a mouse model of osteogenesis imperfecta directly contributed to bone formation, whereas they speculated that the transplanted cells also exerted a paracrine effect to induce host cell osteogenesis [21]. Indeed, a recent study demonstrated that transplantation of osteogenic differentiated MSCs seeded on a collagen membrane induced bone regeneration via donor and host cells osteogenesis in a SCID mouse calvarial defect model [22]. Consistent with this recent report, implantation of C-MSCs generated with xeno-free osteo-inductive medium facilitated bone regeneration via direct and indirect osteogenesis. Various factors, including cell type, degree of cell differentiation, and transplantation procedures may be responsible for the discrepancy in the findings of grafted MSCs actions between previous reports and the present study.

Although Decell-C-MSCs transplantation could provide human COL1 as the scaffold for the host mouse cell infiltration into the bony lesion area, they failed to induce bone matrix reconstruction (Figure 5). These findings clearly suggested that host indirect bone regeneration induced by C-MSCs could be attributed to the paracrine factors produced by transplanted human cells. Indeed, it was reported that grafted MSCs expressed bone morphogenetic protein (BMP)-2 to induce bone regeneration in a mouse bone fracture model [16,23]. Moreover, Zhou et al. demonstrated that vascular endothelial growth factor (VEGF)-A, a robust angiogenic factor, produced by human MSCs pretreated with osteo-inductive medium plays a crucial role in host indirect bone reconstruction in a mouse calvarial defect model [24]. These osteogenic and angiogenic paracrine factors produced by transplanted human cells may be responsible for the host indirect osteogenesis induced by C-MSCs transplantation.

On the other hand, there is a possibility that host mice cells were also associated with donor human cells direct osteogenesis in the process of bone regeneration induced by C-MSCs. This is because we found that cuboidal osteoblastic mice cells lined the grafted C-MSCs at 2 weeks and after that human bone-related proteins deposition and osteocytes differentiation appeared (Figure 4). It is widely accepted that osteoblasts are responsible for the secretion of various kinds of osteogenic factors and the deposition of mineralized matrix to facilitate the bone formation [25]; therefore, host mice osteoblastic cells may facilitate osteogenic differentiation of grafted human donor cells. Taken together, these findings indicated that donor and host cells act in a coordinated manner to accomplish successful bone regeneration following the transplantation of C-MSCs, although additional studies investigating the paracrine factors from donor and host cells are needed.

There are a number of studies developing bone regenerative therapy by using MSCs. However, most of them employed artificial scaffolds to graft cells into the defect area. Even though promising biocompatible materials that facilitate bone regenerative properties in the transplanted cells are becoming established [26,27], their use may impose two different roles on the grafted (or host) cells to induce successful bone regeneration: “generating new bone” and “metabolizing the artificial material”. These two discrete roles may be a burden on the cells and result in delayed tissue regeneration. Transplantation procedures for C-MSCs that can graft the cells using self-produced COL1 as the scaffold seems to have a competitive advantage in this respect. It is well accepted that COL1 is an essential matrix protein for bone formation [28], the grafted (or host) cells can avoid the necessity of metabolizing any artificial material and exercise their biological role in the formation of physical new bone. In fact, grafted C-MSCs showed direct osteogenesis, as shown by bone-related matrix protein deposition, osteocyte differentiation, and new bone formation that is similar to physical membranous ossification. If there are artificial materials, this physical bone reconstruction process caused by grafted cells may be disrupted.

For transplantation therapy with C-MSCs, however, larger bone defect cases in clinical orthopedics could still be a challenge. Even though we applied forty-eight C-MSCs into a beagle dog periodontal defect model to induce successful tissue regeneration [9], a segmental tibial fracture will require the implantation of approximately 200–300 C-MSCs. It is unclear if all transplanted C-MSCs remain in such a large defect area to exert their osteogenic effects. Subsequently, in order to graft more than several hundred C-MSCs appropriately into larger damaged tissue, combined use of some artificial materials that will not disrupt the osteogenic property of C-MSCs may be needed.

Regarding this point, 3D cell-scaffold constructs generated with bioreactor could be helpful. Since it requires the artificial scaffold, there is a disadvantage associated with biodegradability as described above. However, bioreactor systems can sustain dynamic biological processes under well-controlled culture condition [29]. Accordingly, it can provide larger and more functional tissue constructs consist of various type of cells than C-MSCs. Indeed, Lin et al. generated tissue construct consists of chondral and osseous tissue by using MSCs and bioreactor, which could be supportive to treat the critical size of bone defect [30]. Moreover, recent studies demonstrated bioreactor can provide vascularized bone-like constructs [31]. Taken together, combined usage of 3D cell-scaffold constructs generated with bioreactor and scaffold-free C-MSCs transplantation system will cover the each other’s shortcomings, and thereby could be a promising cell therapy for the severe bone defect cases in clinical orthopedic.

A decade ago, FBS was frequently employed to maintain and expand MSCs for cell transplantation therapy. Indeed, Lalu et al. reported that 27 out of 36 clinical studies used FBS for human MSCs culture [32]. Recently, however, various side effects or risks related to the utilization of animal-derived products for the development of MSCs therapy have been recognized. Patient self-serum could be an alternative to FBS, though its isolation should increase the burden on patients. Accordingly, xeno-free and serum-free culture medium has attracted medical and scientific attention. To develop safe and reliable transplantation therapy using C-MSCs, we employed commercially available xeno-free medium. The C-MSCs generated using xeno-free medium exerted bone regenerative properties similar to C-MSCs prepared with FBS [8,10,33]. More importantly, we reported previously that C-MSCs can be cryopreserved with xeno-free cryoprotectant [33]. In addition, we also demonstrated that C-MSCs treated with IFN-γ may be applicable for clinical allogenic bone regenerative therapy due to its highly regulated immunomodulatory properties [10]. Taken together, the combination of cryopreservation, IFN-γ pretreatment, and xeno-free medium may lead to reliable “off-the-shelf” C-MSCs therapy for bone regeneration.

## 4. Materials and Methods

### 4.1. C-MSC Preparation and Culture

C-MSCs were prepared as reported previously with minor modifications [34]. Briefly, human bone marrow MSCs provided by RIKEN BIORESOURCE CENTER (Tsukuba, Ibaraki, Japan) were seeded at a density of 1.0 × 10^5^ cells/well in 48-well plates (Corning, Corning, NY, USA) and cultured with Prime-XV MSC expansion XSFM (Irvine Scientific, Santa Ana, CA, USA) for 4 days. To obtain C-MSCs, confluent cells that had formed on the cellular sheet, consisting of the ECM produced by MSCs themselves, were scratched by using a micropipette tip and then torn off. The C-MSCs detached from the bottom of the plate in a sheet shape were transferred to a 24-well ultra-low-binding plate (Corning) and rolled up to make a round clump of cells. The cell clumps were maintained in MSCgo Osteogenic-SF, XF medium (Biological Industries, Beit Haemek, Israel) for 3 days (Figure 1A).

### 4.2. Flow Cytometric Analysis of MSC Markers

To investigate whether C-MSCs generating process with xeno-free condition impacts MSCs biological property, FACS analysis was conducted. Briefly, cells cultured with Prime-XV MSC expansion XSFM for 4 days were treated with accutase (Innovative Cell Technologies, San Diego, CA, USA) at 37 °C for 20 min. The dissected samples were filtered through sterile 70 µm nylon cell strainers (BD Biosciences, Franklin Lakes, NJ, USA) to obtain cell suspensions. The cells were then incubated with antibodies listed in Supplemental Table S1 for 1 h at room temperature. The expression profile of each molecule was determined using a FACScan flow cytometer (BD Biosciences) with Cell Quest software (BD Biosciences).

### 4.3. Decellularization of C-MSCs

To generate decellularized ECM derived from C-MSCs (Decell-C-MSCs), freeze-thaw cycling with ammonium hydroxide (NH_4_OH) was employed as reported previously [35]. Briefly, C-MSCs were washed with PBS and MilliQ water. The cell clumps were frozen at −80 °C, thawed at room temperature, and washed with MilliQ water. This freeze-thaw cycle was repeated four times. Then, the specimens were immersed in 25 mM of NH_4_OH aqueous solution for 1 h and washed with MilliQ water six times.

### 4.4. Histological and Immunofluorescence Analysis of C-MSCs and Decell-C-MSCs

C-MSCs or Decell-C-MSCs were fixed with 4% paraformaldehyde in PBS. The samples were embedded in paraffin. Eight-micrometer-thick sections were prepared. The samples were then stained with hematoxylin and eosin (H&E) and observed using a light microscope.

Regarding immunofluorescence analysis, the fixed specimens were embedded in Tissue-Tek OCT compound (Sakura, Torrance, CA, USA), and 20-μm-thick serial sections were cut using a cryostat. The sections were washed with PBS and then non-specific binding was blocked with 5% BSA/0.1% TWEEN 20/ PBS blocking solution. These sections were immunostained with the antibodies listed in Supplemental Table S2. Then, the nuclei were counterstained with 4’,6-diamidino-2-phenylindole (DAPI) (Invitrogen, Carlsbad, CA, USA, 5 μg/mL). After washing the samples with PBS, we detected the fluorescence signals using an Olympus FV1000D laser scanning confocal microscope (Olympus, Tokyo, Japan).

To detect apoptotic cells, the sectioned samples were assessed using a DeadEnd^TM^ Fluorometric TdT-mediated dUTP nick end labeling (TUNEL) System (Promega, Madison, WI), in accordance with the manufacturer’s instructions. Fluorescence signals were detected using an Olympus FV1000D laser scanning confocal microscope.

To visualize denatured COL1, 5-FAM-conjugated Collagen Hybridizing Peptide (F-CHP) (3Helix, Inc., Salt Lake City, UT, USA) was used in accordance with the manufacturer’s instructions. Briefly, sectioned samples were incubated with 15 µM F-CHP in PBS solution at 4 °C overnight. After washing the samples, fluorescence signals were detected by using an Olympus FV1000D laser scanning confocal microscope.

### 4.5. Surgical Procedures

To assess the bone regenerative properties of C-MSCs and Decell-C-MSCs, seventy two male NOD/SCID mice (7–8 weeks old) (Charles River Laboratories Japan, Yokohama, Japan) were employed as a calvarial defect model after approval had been obtained from the Animal Care Committee of Hiroshima University (protocol number: A12-58 and A13-165, 11 May 2018). The animals were anesthetized with an intraperitoneal injection of 20% ethyl carbamate (30 mg/kg body weight). The skin at the surgical site was shaved and disinfected, and then a sagittal skin incision was made from the occipital to the frontal bone. The skin flap, including the periosteum, was then dissected and elevated. Avoiding the cranial suture, calvarial defects of 1.6 mm diameter was created in the parietal bone. C-MSCs or Decell-C-MSCs were grafted into the defect with no artificial scaffold. In addition, no implant group was included as a control (*n* = 6/group for 1, 2, 4, and 8 weeks observation, respectively).

### 4.6. Micro-CT Analysis

The animals were sacrificed at 1, 2, 4, and 8 weeks after surgery, and the cranial region was scanned by using a SkyScan1176 in vivo μCT (Bruker, Billerica, MA, USA) with following conditions: 50 kV, 0.5 mA, 8 μm pixel size, and 0.5 degree rotation step with 230 ms exposure time. Three-dimensional reconstructions were generated using CTVOL software 2.3.2.0 (64bit) (Bruker). The region of interest (ROI) for bone volume measurement was the 1.6-mm circle of the bone defect that consists of 33 2D slices (approximately 600 μm thickness). Segmentation of the ROI and following bone volume measurement were performed by CT-An software 1.12.0.0+ (Bruker) with a threshold range of 80–255 [36].

### 4.7. Tissue Preparation and Histological Analysis

Mice were sacrificed at 1, 2, 4, and 8 weeks after surgery. Calvarial bones were collected, fixed with 4% paraformaldehyde overnight, and decalcified with 10% ethylenediaminetetraacetic acid (pH 7.4) for 10 days. After decalcification, the samples were dehydrated through grade ethanol, cleared with xylene, and embedded in paraffin. Semi-serial sections (8 μm) were cut in the frontal plane. These sections showing the central portion of the bone defect were then stained with H&E or AZAN and observed using light microscopy.

To detect human vimentin, COL1, OPN, and OCN, immunofluorescence analysis was conducted. Briefly, the semi-serial sections (20 μm) were incubated in LAB solution (Polyscience, Warrington, PA, USA) for 15 min at room temperature to activate antigens and were blocked with 5% BSA/0.1% Tween/PBS blocking solution at room temperature for 1 h. For OCN staining, before this blocking, the samples were pretreated with Mouse on Mouse blocking reagent (Vector Laboratories, Burlingame, CA, USA) at room temperature for 1 h. These specimens were then immunostained with the antibodies listed in Supplemental Table S2. To remove the autofluorescence in tissue sections, the samples were treated with Vector^®^ TrueVIEW Autofluorescence Quenching kit (Vector) for 5 min at room temperature. Then, the nuclei were counterstained with DAPI (Invitrogen, 5 μg/mL). After washing the samples with PBS, we detected fluorescence signals using an Olympus FV1000D laser scanning confocal microscope.

To visualize F-actin filaments, phalloidin staining and 3D confocal imaging was conducted. Briefly, the fixed specimens were embedded in Tissue-Tek OCT compound, and 20-μm-thick serial sections were cut using a cryostat. The sections were washed with PBS and then non-specific binding was blocked with 5% BSA/0.1% TWEEN 20/ PBS blocking solution. These sections were incubated with a rabbit anti-human vimentin IgG antibody (clone SP20, abcam, 1:100) at 4 °C overnight. After being washed 3 times with PBS, samples were incubated for 2 h with an Alexa Fluor 488^®^ goat anti-rabbit IgG antibody (1:100; Invitrogen) and Alexa Fluor 594^®^ phalloidin (Invitrogen, 1:50) at room temperature. Then, a z-axis series of fluorescence images was obtained using an Olympus FV1000D laser scanning confocal microscope. Forty optical serial z-axis sections (0.5 µm thick) were obtained and reconstructed in 3D using FV10-ASW image software 04.02.03.06 (Olympus, Tokyo, Japan).

### 4.8. Statistical Analysis

Statistical analysis was performed using two-tailed unpaired Student’s *t*-test to compare two groups or by one-way ANOVA with Tukey–Kramer post hoc test to compare three groups. Any *p* values less than 0.05 were considered to be significant.

## 5. Conclusions

In conclusion, we established C-MSCs in xeno-free and serum-free conditions. The transplantation of C-MSCs with no artificial scaffold successfully induced bone regeneration in a SCID mice calvarial defect model. In addition, bone regeneration induced by C-MSCs was due to donor direct and host indirect osteogenesis. These findings suggested that C-MSCs could be a safe and reliable scaffold-free cell therapy for clinical bone destructive diseases.

## Supplementary Materials

Supplementary materials can be found at https://www.mdpi.com/1422-0067/20/16/3970/s1.
