# Supplementary material for: Clumps of Mesenchymal Stem Cell/Extracellular Matrix Complexes Generated with Xeno-Free Conditions Facilitate Bone Regeneration via Direct and Indirect Osteogenesis
Source: Int J Mol Sci. 2019 Aug 15;20(16):3970. doi: 10.3390/ijms20163970 (PMC6720767; doi:10.3390/ijms20163970)
Supplement: Supplementary file 1 [file ijms-20-03970-s001.pdf]

Table S1. Antibodies for FACS.

Antigen	Antibody Type	Clone	Conjugate	Source	Cat. No.	Dilution
CD105	Mouse IgG1, k	Mono (SN6)	PE	eBioscience	12-1057-41	1/100
CD90	Mouse IgG1, k	Mono (5E10)	PE	BD Pharmingen	561970	1/100
CD73	Mouse IgG1, k	Mono (AD2)	PE	BD Pharmingen	550257	1/100
CD45	Mouse IgG1, k	Mono (HI30)	PE	BD Pharmingen	555483	1/100
CD34	Mouse IgG1, k	Mono (563)	PE	BD Pharmingen	550781	1/100
None	Mouse IgG1, k	Mono (MOPC-21)	PE	BD Pharmingen	559320	1/100

Table S2. Antibodies for immunostaining

Antigen	Host Species	Clone	Conjugate	Source	Cat. No.	Dilution
Human vimentin	Rabbit	Mono (SP20)	None	abcam	ab16700	1/100
Human COL1	Rabbit	Mono (EPR7785)	None	abcam	ab138492	1/100
Human OPN	Goat	Poly	None	R&D	AF1433	1/100
Human OCN	Mouse	Mono (OCG3)	None	abcam	ab13420	1/100
Mouse IgG(H+L)	Goat	Poly	Alexa 594	ThermoFisher	A11032	1/100
Rabbit IgG(H+L)	Goat	Poly	Alexa 488	ThermoFisher	A11008	1/100
Goat IgG(H+L)	Chicken	Poly	Alexa 594	ThermoFisher	A21468	1/100
